# Relationship between Eccentricity and Volume Determined by Spectral Algorithms Applied to Spatially Registered Bi-Parametric MRI and Prostate Tumor Aggressiveness: A Pilot Study

**DOI:** 10.3390/diagnostics13203238

**Published:** 2023-10-17

**Authors:** Rulon Mayer, Baris Turkbey, Peter L. Choyke, Charles B. Simone

**Affiliations:** 1 Department of Radiation Oncology, Perelman School of Medicine, University of Pennsylvania, Philadelphia, PA 19104, USA; 2 Oncoscore, Garrett Park, MD 20896, USA; 3 National Institutes of Health, Bethesda, MD 20892, USA; ismail.turkbey@nih.gov (B.T.); pchoyke@mail.nih.gov (P.L.C.); 4 New York Proton Center, New York, NY 10035, USA; csimone@nyproton.com

**Keywords:** bi-parametric MRI, prostate cancer, spatial registration, tumor morphology, supervised target detection, spectral analysis

## Abstract

(1) Background: Non-invasive prostate cancer assessments using multi-parametric MRI are essential to the reliable detection of lesions and proper management of patients. While current guidelines call for the administration of Gadolinium-containing intravenous contrast injections, eliminating such injections would simplify scanning and reduce patient risk and costs. However, augmented image analysis is necessary to extract important diagnostic information from MRIs. Purpose: This study aims to extend previous work on the signal to clutter ratio and test whether prostate tumor eccentricity and volume are indicators of tumor aggressiveness using bi-parametric (BP)-MRI. (2) Methods: This study retrospectively processed 42 consecutive prostate cancer patients from the PI-CAI data collection. BP-MRIs (apparent diffusion coefficient, high b-value, and T2 images) were resized, translated, cropped, and stitched to form spatially registered BP-MRIs. The International Society of Urological Pathology (ISUP) grade was used to judge cases of prostate cancer as either clinically significant prostate cancer (CsPCa) (ISUP ≥ 2) or clinically insignificant prostate cancer (CiPCa) (ISUP < 2). The Adaptive Cosine Estimator (ACE) algorithm was applied to the BP-MRIs, followed by thresholding, and then eccentricity and volume computations, from the labeled and blobbed detection maps. Then, univariate and multivariate linear regression fittings of eccentricity and volume were applied to the ISUP grade. The fits were quantitatively evaluated by computing correlation coefficients (R) and *p*-values. Area under the curve (AUC) and receiver operator characteristic (ROC) curve scores were used to assess the logistic fitting to CsPCa/CiPCa. (3) Results: Modest correlation coefficients (R) (>0.35) and AUC scores (0.70) for the linear and/or logistic fits from the processed prostate tumor eccentricity and volume computations for the spatially registered BP-MRIs exceeded fits using the parameters of prostate serum antigen, prostate volume, and patient age (R~0.17). (4) Conclusions: This is the first study that applied spectral approaches to BP-MRIs to generate tumor eccentricity and volume metrics to assess tumor aggressiveness. This study found significant values of R and AUC (albeit below those from multi-parametric MRI) to fit and relate the metrics to the ISUP grade and CsPCA/CiPCA, respectively.

## 1. Introduction

Accurate and reliable assessments of patients with prostate cancer are essential for properly managing this highly prevalent and potentially lethal disease [1]. Conventionally, the prostate is sampled with biopsy needles [2] with the aid of an ultrasound or MRI [3] and then processed and examined by a pathologist [4]. Biopsies are uncomfortable for the patient and can lead to side effects such as infection, pain, and hemorrhage [5]. In addition, possible incorrect placement of the needle [6] can lead to false negatives or underreport the aggressiveness of the disease. To reduce the deficiencies in this approach, prostate tumor assessments now routinely include a non-invasive multi-parametric MRI with analysis of the images using the Prostate Imaging Reporting And Data System (PI-RADS) [7]. PI-RADS relies on the subjective assessment of radiologists to properly identify and classify lesions. Despite training and education, expertise varies among different radiologists [8] due to the subjective character of the assessment. Instead, to achieve more consistent results, quantitative approaches using an artificial intelligence (AI) program have been applied to MRI [9]. An AI program computes textural features derived from the spatial analysis of the outlined regions of interest denoting tumors. The AI program filters the large number of features (hundreds to thousands) and, following training, combines them to predict which lesions warrant a biopsy. Such an approach requires large training sets, and the results are limited by restricted clinical conditions that govern the training sets.

More recently [10,11,12,13,14,15,16], a statistical spectral approach has been applied to spatially registered multi-parametric MRIs. This approach was adapted from analyzing hyperspectral cubes, such as in remote sensing, defense and security applications, and environmental studies. Instead of a hypercube generated from dispersive lenses and push-broom sensors, this approach resizes the MRIs to a common spatial resolution, translates them, crops them to form a multi-spectral cube, and stitches the cubes to depict the entire three-dimensional structure. Each voxel is a three-dimensional vector whose components are the values in the bi-parametric MRI space (T2, ADC, High B Value). A tumor is characterized as a three-dimensional signature vector that differs from the mean background (normal prostate) vector. Second order statistics (co-variance matrix, mean vector) characterize the background (normal prostate).

The earliest [10,11,12,13,14,15] spectral statistical studies examined multi-parametric MRIs of 26 prostate cancer patients from the National Institutes of Health. That data set included the analysis of images using dynamic contrast enhancement techniques that depict the tumor vasculature and summarize the evolution time of the contrast material. This earlier work found that prostate tumor signatures could successfully characterize prostate tumors and be transformed, and that the signal to clutter ratio and tumor volume correlate with the Gleason score, which is a measure of prostate tumor aggressiveness. Similarly, the tumor shape (eccentricity) is negatively correlated with tumor aggressiveness.

To further minimize patient discomfort, reduce the possibility of side effects, simplify the clinical setup, minimize the scanning time [17], and reduce financial burden [18], there is increasing interest in assessing patients using MR scans without injecting a contrast material. This is referred to as bi-parametric MRI (BP-MRI) and is composed of T2 images and diffusion weighted MRI scans, such as a high b-value scan and an apparent diffusion coefficient (ADC) map [16]. Although clinically convenient, the reduction in clinical information derived from the dynamic contrast enhancement (DCE) technique [19], specifically with respect to tumor vasculature, is expected to reduce the predictive power of spectral features studied in previous work. However, in the clinical realm, there is less reliance on DCE MRI than previously [9,17,18]. In the case of signal to clutter ratio, its correlation with tumor aggressiveness is strong and is, perhaps, sufficient. The current study examines whether tumor volume and eccentricity is correlated with prostate tumor aggressiveness using bi-parametric MRI.

## 2. Materials and Methods

### 2.1. Overview

**Figure 1:**
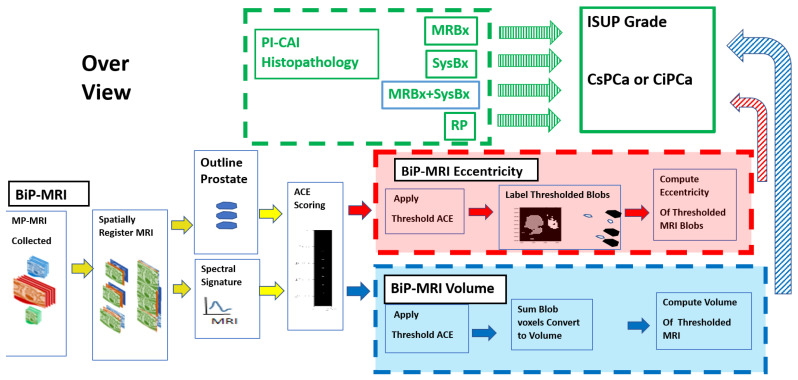
The schematic depicts assembly, relationship, and arrangement of spatially registered cubes, tumor signature, and normal prostate mask input for ACE detection calculations, thresholding, masking and prostate tumor eccentricity and volume calculations, and linear regression and logistic probability fits. MRBx, SysBx, MRBx + SysBx, and RP were used in the PI-CAI histopathology assessment. Direction of output data to be used as input denoted by arrows. Red arrows and box indicate eccentricity calculations from bi-parametric MRI-based data; blue arrows and box denote tumor volume estimate from bi-parametric MRI.

Figure 1 schematically shows how this study compared metrics related to prostate tumor aggressiveness. Specifically, the International Society of Urological Pathology (ISUP) grade [20] clinically defines cases of prostate cancer as either clinically significant prostate cancer (CsPCa where ISUP ≧2) or clinically insignificant prostate cancer (CiPCa where ISUP < 2). The ISUP-defined cases of CsPCa and CiPCa were compared with metrics (the tumor volume [11] and eccentricity [12]) derived from spatially registered BP-MRIs. The patient BP-MRI data in this study were collected and archived as part of the PI-CAI Challenge [21]. Pathology examinations [22] of the histopathology slides were used to determine the ISUP grade. In the BP-MRI part [10,11,12,13,14,15,16], the MRI sequences, specifically the ADC, HBV from the DWI, and T2 images were resized, finely translated, and cropped to create a common spatial resolution and field of view. The individual cubes for each slice were stitched together to depict the entire prostate and surrounding tissues to form a thin hypercube. The normal prostate was manually outlined using the spatially registered hypercube. In-scene signatures were derived from the hypercube. The signatures and masked normal prostate data provide input for the ACE computation [10,11,12,13,14,15,16]. Tumor eccentricity and volume were fitted using a linear (logistical probability) regression analysis to the ISUP grade (CsPCa/CiPCa), respectively. Correlation coefficients (R), *p*-values, the area under the curve (AUC) and receiver operator characteristic (ROC) curve measurements [23] were computed to evaluate the linear regression (logistic probability) fits.

The Python 3 programming language was employed to spatially register and process the bi-parametric data, compute the tumor volume and eccentricity, generate linear regression fits, and determine the ROC curve, AUC, correlation coefficients, and *p*-values.

### 2.2. Study Design and Population

PI-CAI [21] archived the bi-parametric prostate tumor MRI data set. The PI-CAI provides public access to annotated multiple-center, multiple-vendor datasets of 1500 BP-MRI exams that include clinical and acquisition variables. The histopathology techniques range from MRI-guided biopsy (MRBx), systematic biopsy (SysBx), combined MRBX and SysBx (MRBx + SysBx), and radical prostatectomy (RP) [22]. A subset of the 1500-patient cohort underwent or had available biopsy results. Patients were scanned in four centers using nine scanners manufactured by Siemens and Philips [21]. Each centers’ staff pathologists and radiologists assessed patients at their center, independently of each other. The PI-CAI data compilation [21] only includes the bi-parametric MRI, specifically the ADC, HBV, and T2 sequences. 

Table 1 summarizes the clinical data for 42 consecutive patients and biopsy results in the PI-CAI data collection. All patients had biopsy-proven adenocarcinoma of the prostate, with a mean patient age of 65.1 years (range, 50 to 78 years), mean PSA of 13.49 ng/mL (range, 1.5 to 81.95 ng/mL), mean prostate volume mean of 60.6 cm^3^ (range, 19 to 192 cm^3^), and mean ISUP grade of 1.12 (range, 0 to 5) (Table 1). No restrictions were placed on the tumor location within the prostate in this study. All cases were anonymized prior to analysis.

### 2.3. Spatial Registered Hypercube Assembly: MRI Components, Sequences

Structural (T2) images, DWIs, and particularly the ADC and HBV images, composed the bi-parametric MRI data collect [21]. The PI-CAI excluded DCE images.

### 2.4. Spatial Registered Hypercube Assembly, Pre- Image Processing Analysis

Prior to registration, scanning parameters (spatial resolution, offsets for the scanning setup) were read from image header files for all MRI sequences (ADC, HBV, and T2) for each patient. All MRI images were digitally resized [10,11,12,13,14,15,16] to the sequence with the lowest spatial resolution in the transverse direction. The T2 images were translated a few voxels to the reference image (ADC and HBV) using the offsets read from the image header files. The slices were positionally transposed to the offsets based on the known location of the axial offsets. Finer adjustments required additional small transverse translation based on a visual inspection of the T2 image to align with the ADC and HBV image. Stacked individual slices were appropriately scaled, translated, and cropped so as to be spatially registered at the voxel level and thereby constitute a “cube”. After cropping, all image sequences in the stack shared the same field of view (FOV). The “three-dimensional” (two-dimensional slice plus the “spectral” dimension of the spatially registered ADC, HBV, and T2 images) cubes were subsequently “stitched” together into the narrow three-dimensional hypercube that depicts the entire scan including the prostate. The stitching (or mosaicking) resembles the approach employed in remote sensing applications that stitch (mosaic) large areas in order to enhance the visual depiction of the entire imaged area and expedite the speed for processing the high dimensional data. Spatial registration and stitching took a few seconds to process using a Windows 10, Base Speed 2 Ghz, Cache memory 8 Gbyte machine for each patient.

**Figure 2:**
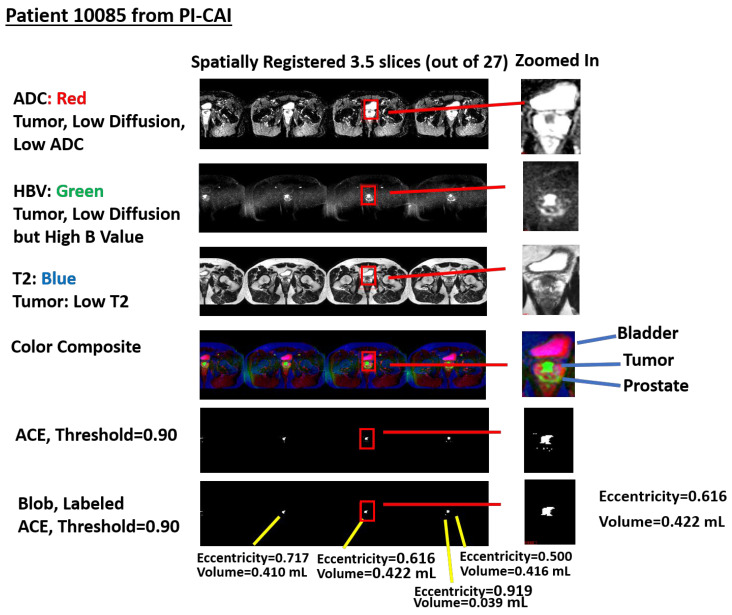
Spatially registered ADC, HBV, T2, and color composite images displayed following assignment to red, green, and blue colors, respectively. Zoomed ADC, HBV, T2, and color composite image also shown. Tumor appears as green; bladder appears as magenta. Also shown are voxels with ACE scores exceeding threshold = 0.90 and blobs and their eccentricity and volume computations.

Figure 2 shows an example of a stitched spatially registered image. Figure 2 shows three spatially registered sequences (ADC, HBV, and T2) and a color composite generated by assigning red, green, and blue to the ADC, HBV, and T2 sequences, respectively. Spatially registered “cubes” were “stitched” together and abutted together in the horizontal direction and are also shown in Figure 2. A shared zoomed-in part of the image is shown for the ADC, HBV, T2, and composite color image. The tumor in this color scheme appears green (low ADC, High B Value HBV, and low T2). Also, Figure 2 shows the voxels that exceed the threshold = 0.90 for ACE along with displays of the blobbing and labeling [12] of voxels exceeding the ACE threshold = 0.90. The computed eccentricities and volumes are listed for every blob. 

### 2.5. Adaptive Cosine Estimator (ACE) Algorithm

Supervised target detection algorithms [10,24] peruse and classify a voxel into either a target (prostate tumor) or background (normal prostate) based on information about the target (tumor), specifically the tumor signature. The tumor signature S is a three-dimensional vector whose components are intensity values from within the manifold (T2, High B Value, ADC) that relate to the target. The background is characterized by a mean three-dimensional vector m and covariance matrix CM (3 dimensions × 3 dimensions) that includes the variance and accounts for correlations among the different dimensions. The Adaptive Cosine Estimator (ACE) algorithm is one supervised target detection algorithm [10,24]. A multi-dimensional cone surrounding the target signature S describes the ACE decision surface. Voxels whose ACE scores lie within the decision cone are assigned to the target, whereas those outside the cone are assigned to the background. Appendix A.1. offers a more detailed summary of the ACE algorithm and provides clarifying equations.

### 2.6. Tumor Volume Measurements, Supervised Target Detection

The ACE algorithm was applied to the spatially registered BP-MRI [11] to generate a metric associated with the tumor volume. Voxels that lie inside the decision cone or exceed a threshold for ACE scores were assigned to the tumor. Normal tissue was assigned to voxels that resided outside the decision cone or had ACE scores residing below the threshold. The number of voxels that exceeded the threshold (tumor) were counted. This sum is converted to volume based on the MRI spatial resolution. Appendix A.2. summarizes some of the mathematics behind the tumor volume computation. For more details, see Reference [11].

### 2.7. Labeling and Blob Generation

In the computer vision field, blobbing and labeling [12,25] means aggregating neighboring voxels in an objective way. The blobbing is applied to a mask image or binary image after the application of a threshold to the ACE detection image. The value of 1 or 0, or “True” or “False”, is associated with the tumor (background) in each masked image. Blobbing is based on whether the voxels that are associated with the tumor form an 8-pixel connected neighborhood. Each “True” voxel peruses voxels within a given neighborhood (1 voxel away) to see if they are also “True”. If they are “True” they are connected, collected, and labeled as a member of a blob. Blobs smaller than <5 voxels (~10^−2^ mL) were filtered out.

### 2.8. Eccentricity Calculation

The eccentricity [12] for every labeled blob was calculated using custom software that was coded in Python 3. After collecting a blob, the moment of inertia matrix I for the kth blob was computed. The eigen equation was solved for the moment of inertia I and the eigenvalues for each blob was determined. The largest eigenvalue was assigned to the large axis l_k_, and the second eigenvalue was assigned to the transverse moment s_k_. The eccentricity E_k_ for the kth blob is a normalized difference of the major axis and minor axis. Eccentricity values E_k_ range from 0 (spherical shape) to 1 (line). Appendix A.3. offers a more detailed summary of the eccentricity computation and provides clarifying equations.

### 2.9. Univariate and Multivariate Fitting

Using a univariate and multivariate linear regression analysis [26], one or multiple independent variables are fitted to a single independent variable. In this study, the independent variables correspond to the largest blob’s eccentricity and largest and average blob’s volume generated after applying varying ACE thresholds. The dependent variable is the ISUP grade or CsPCa. The latter is a categorical variable (binary variable or either True or False). The fits minimize the error in a least squares calculation by finding the optimal fitting coefficient for the independent variable. These fitting coefficients can be applied to the independent variables to generate a fit, and then compared to actual data. Correlation coefficients and fitted lines test the agreement of the computed p values to assess the probability that the fit is or is not correlated. Confidence Intervals are computed for every variable along with p values for the multivariable fit. Appendix A.4. offers a more detailed summary of the linear regression fitting and provides clarifying equations.

### 2.10. Logistic Regression

A logistic regression analysis [27] fits the individual or multiple independent variables (specifically, the processed tumor volume, tumor eccentricity, or clinical patient data) to the dependent categorical variable, CsPCa, that takes only two values. Clinically significant prostate cancer (CsPCa) was associated with ISUP grade ≥ 2, and clinically insignificant prostate cancer (CiPCa) was associated with ISUP < 2. The training/test sets were randomly assigned among the 42 patients. The training/test sets retained a 70%/30% ratio for 1000 different random sets for all patients and generated a distribution of ROC [23] curves and associated AUC scores. A histogram summarizing the distribution of AUC scores was computed, along with the 2.5% and 97.5% AUC scores, in order to generate the 95% confidence interval. The AUC scores and the 95% confidence interval from the ROC curves assessed the fit quality.

### 2.11. Receiver Operator Characteristic

The receiver operator characteristic (ROC) curve [23] is a graphical way of evaluating a binary classifier. The binary classifier in this study distinguishes CsPCa (ISUP ≥ 2) from CiPCa (ISUP < 2). The ROC curve plots the true positive or sensitivity (probability of accurately detecting CsPCa) against the false positive rate or 1-specificity rate, where sensitivity corresponds to accurately detecting CiPCa. The ROC curve is a monotonically increasing function. A way to summarize and assess the binary classifier is to compute the area under the curve (AUC) for the ROC curve. The AUC varies from 0 (poor performance) to 1 (perfect classifier). Appendix A.5. offers a more detailed summary of the ROC curve considerations and provides clarifying equations.

## 3. Results

### 3.1. Univariate Fits

**Figure 3:**
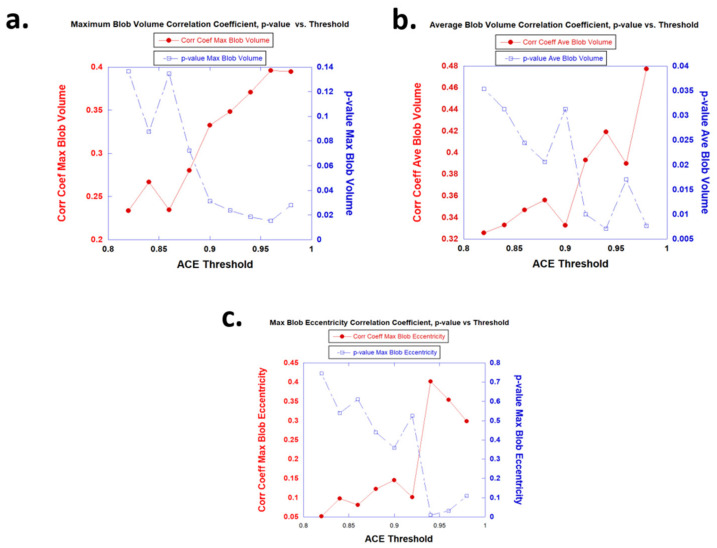
Plot of correlation coefficients (R) (red) and *p*-values (blue) from linear regression fitting of maximum blob volume (**a**), average blob volume (**b**), and maximum blob eccentricity (**c**) to the ISUP.

Figure 3 shows plots of correlation coefficients (R) (red) and *p*-values (blue) from the linear regression fitting of the maximum blob volume, average blob volume, and maximum blob eccentricity for Figure 3a–c, respectively, to the ISUP grade for 42 patients as a function of the ACE thresholds. A higher (lower) correlation coefficient (*p*-value) corresponds to high ACE thresholds (>0.90). The correlation coefficients (*p*-values) generally monotonically increase (decrease) as a function of the ACE threshold. Sufficiently low *p*-values (<0.05) are only observed for the ACE threshold (0.90).

Table 2 summarizes the results of fitting the correlation coefficients (R) and *p*-values from the linear regression fitting of maximum blob volume, average blob volume, and maximum blob eccentricity to the ISUP grade (for the denoted ACE thresholds) for 42 patients. In addition, Table 2 records the evaluation of logistic probability fits of maximum blob volume, average blob volume, and maximum blob eccentricity to the CsPCa (ISUP ≥ 2), namely the AUC from the ROC curves with 95% confidence intervals. Also shown are the coefficient correlation, *p*-values, and AUC for the univariate (logistic probability) fitting to the ISUP grade (CsPCa) using clinical parameters (prostate serum antigen (PSA), prostate volume, patient age). The R, *p*-values, and AUC values using the clinical parameters are lower than the better performing tumor eccentricity and volume (ACE threshold > 0.92).

**Figure 4:**
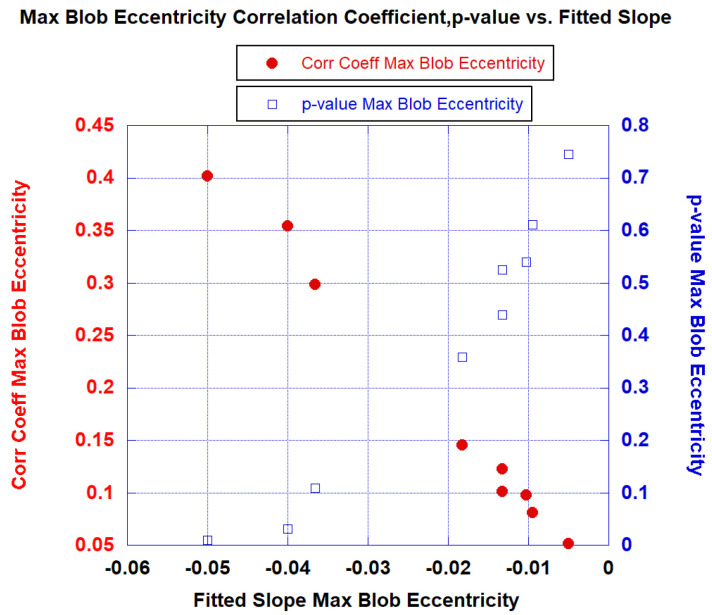
Plot of correlation coefficient (red) and *p*-values (blue) against the fitted slops from fitting the maximum blob eccentricity to ISUP grade.

Figure 4 plots the correlation coefficients (red) and *p*-values (blue) against the fitted slopes from fitting the maximum blob eccentricity to the ISUP grade. The steeper the slope, the higher (lower) the correlation coefficient (*p*-value) and further confirming that maximum blob eccentricity is negatively correlated with tumor aggressiveness.

### 3.2. Multivariate Fits

**Figure 5:**
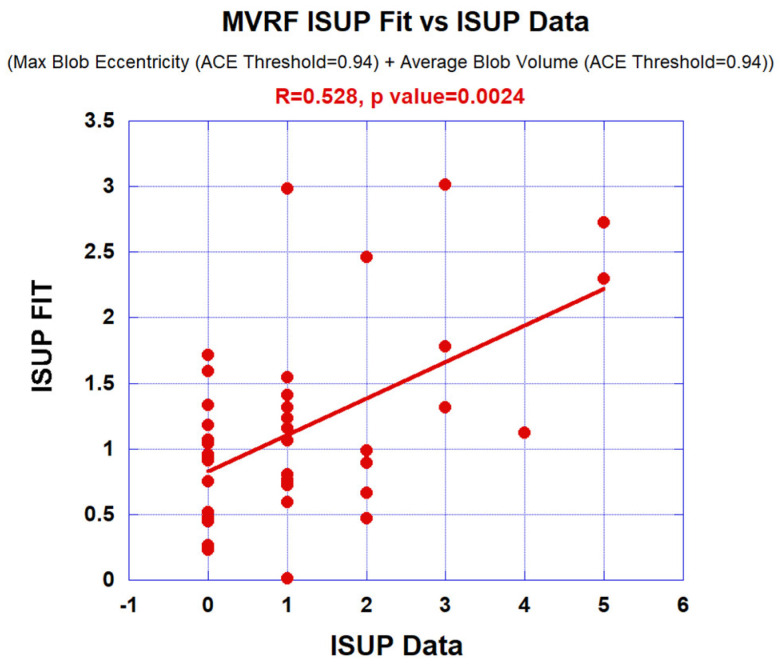
Multivariate fitting (maximum blob eccentricity and average blob volume using ACE Threshold = 0.94) to ISUP grade. Straight line depicts the linear fit.

Figure 5 is one example showing the multivariate fitting (in this case maximum blob eccentricity and average blob volume using ACE Threshold = 0.94) to the ISUP grade. A straight line depicts the linear fit.

Table 3 summarizes multivariate (logistic probability) fits of tumor eccentricity and tumor volume to the ISUP grade (CsPCa) for ACE thresholds of 0.90 to 0.96. Correlation coefficients (R1, R2, R12) and associated *p*-values are shown. The AUC scores with a 95% confidence interval for the multivariate logistic probability fits to CsPCa are shown. Combining the tumor eccentricity and volume in the fitting routine results in larger (smaller) correlation coefficients (*p* values) relative to univariate fits (Table 2).

## 4. Discussion

This study examined the relationship between prostate tumor eccentricity and volume derived from supervised target detection algorithms applied to spatially registered bi-parametric MRI, and tumor aggressiveness. There is a correlation (R~0.30, *p* < 0.05) between the largest blob’s eccentricity and volume as well as the average blob volume, and the ISUP grade. Similarly, the same features used to predict CsPCA resulted in an AUC score of ~0.70. These correlation coefficients and AUC scores are lower than those using the signal to clutter ratio, which were previously [16] applied to the same data set (R~0.5, AUC~1.0), but better than from conventional clinical variables (PSA, prostate volume, age). The R and AUC scores are also lower for bi-parametric MRI relative to the values found from analyzing MRIs [13] that included dynamic contrast enhancement.

The lower correlation coefficients and AUC scores for the ISUP grade and CsPCA multivariate and logistic regression fitting in the bi-parametric MRI study, relative to the earlier MP-MRI study [13], indicates that the images derived from the dynamic contrast enhancement technique actually play a crucial role in delineating the tumor. It appears that the vasculature provides important information regarding the tumor shape. Previous studies of eccentricity [12] and volume [11] used seven components, versus the present study that used only three. This reduction in the number of dimensions means a lower signal to clutter ratio and possibly a reduced discrimination and depiction of the prostate tumor eccentricity and volume.

Although the correlation of eccentricity with tumor aggressiveness is lower for the spatially registered bi-parametric MRI, it is nonetheless negative. That is, the more spherical the maximum blob eccentricity, the more aggressive the prostate tumor. Figure 3 shows that the higher (lower) the correlation coefficient (*p*-value), the more negative the slope was in the univariate fitting. Other studies [28] that employ artificial intelligence have found that shape features are negatively correlated with tumor aggressiveness. Previous work [12] found that eccentricity, derived from a quantitative analysis of tumors outlined by pathologists for stained wholemount prostatectomy slides, negatively correlated with the Gleason score. As was previously noted, prostate tumors are usually adenocarcinomas in the prostate. Similarly, other morphology studies found that adenocarcinomas in breast [29] and lung tissues [30,31] showed that eccentricity negatively correlated with grade.

This manuscript only reports a subset of calculations that were performed for tumor eccentricity and volume. Additional calculations were generated, such as weighted eccentricity, average eccentricity, and total volume. However, these additional calculations correlated poorly with ISUP grade, unlike the calculations for the MP-MRI, and were, therefore, not reported in this manuscript. Similarly, additional processing for the ACE computation, such as removing noisy principal components and regularizing the covariance matrix, failed to achieve high correlation with the ISUP grade and CSPCa, unlike earlier studies that employed these covariance matrix enhancements to elevate the signal to clutter ratio connection [13,14,16].

The determination of the appropriate threshold for ACE detection is an important parameter for applying the masks required in the labeling and blobbing operations [11,12] and, therefore, also for tumor eccentricity [12] and volume calculations [11]. By computing the correlation coefficients from the linear regression analysis with the ISUP grade using varying thresholds, the threshold that resulted in the highest correlation coefficient denoted the optimal threshold. It should be observed that the optimal thresholds (~0.90) for bi-parametric MRI were considerably higher than the previously observed optimal threshold (~0.60) for the MP-MRI [11,12].

To assess the relationship between tumor eccentricity and volume to tumor aggressiveness, this study only reported computations of correlation coefficients (R, R12), *p*-values, F-statistic probabilities, AUC scores, and the 95% confidence intervals. Previous work [12,13,14] on evaluations of prostate cancer patients who were scanned using multi-parametric MRI that included the DCE technique, reported more extensive computations, such as student t-values (with 95% confidence intervals), fitting coefficients (with 95% confidence intervals), D’Agostino–Pearson Omnibus residual normality tests, Shapiro–Wilks residual normality tests, Brausch–Pagan F-values and their *p*-values. The added computations in previous work [12,13,14] confirmed the conclusions derived from those inferred from the correlation coefficient *p*-values alone and added little value to the manuscript. Similarly, although they were computed but not reported in this paper, the additional assessments did not alter the essential conclusions. Not reporting these additional assessments simplified the discussion.

While this study is the first of its kind, it is not without limitations. This work only analyzed a limited number of patients. Consecutive patients from the database were processed in an attempt to minimize bias. Nevertheless, this work should be treated as a pilot study. Additionally, this study did not apply an AI algorithm to the same patient cohort, which means that questions of the relative merits of spectral approaches vs. AI approaches are valid, and therefore could be the subject of future studies.

## 5. Conclusions

In the first study to date on spatially registered BP-MRI, tumor eccentricity and volume was found to correlate with tumor aggressiveness, although the correlation coefficients and AUC scores were lower than those using tumor eccentricity and volume for multi-parametric MRI (that included the dynamic contrast enhancement technique), higher than using conventional clinical variables, and lower than applying the signal to clutter ratio to BP-MRI.

## Figures and Tables

**Table 1:** Summary of patient characteristics.

Clinical Features	Median [Minimum, Maximum]
Age (Years)	65.14 [50.00, 78.00]
PSA Median (ng/mL)	13.49 [1.50, 81.95]
Prostate Volume Median (mL)	60.6 [19.00, 192.00]
**ISUP Grade**	**Patient #**
0	17
1	14
2	5
3	3
4	1
5	2

Abbreviations: PSA: Prostate Serum Antigen; ISUP: International Society of Urological Pathology.

**Table 2:** Summary of univariate fitting.

Univariate Variable	ACE Treshold	R	*p*-Value	AUC [95% CI]
Ave Blob Vol (mL)	0.90	0.333	0.0313	0.641 [0.167–1.0]
Max Blob Vol (mL)	0.90	0.333	0.0313	0.892 [0.667–1.0]
Max Blob Eccentricity	0.90	0.145	0.358	0.466 [0.0–0.917]
Ave Blob Vol (mL)	0.92	0.393	0.010	0.641 [0.167–1.0]
Max Blob Vol (mL)	0.92	0.348	0.0238	0.959 [0.80–1.0]
Max Blob Eccentricity	0.92	0.100	0.525	0.500 [0–0.917]
Ave Blob Vol (mL)	0.94	0.419	0.00712	0.701 [0.333–1.0]
Max Blob Vol (mL)	0.94	0.371	0.0185	0.949 [0.778–1.0]
Max Blob Eccentricity	0.94	0.402	0.0102	0.896 [0.630–1.0]
Ave Blob Vol (mL)	0.96	0.390	0.0171	0.0 [0.0–0.0]
Max Blob Vol (mL)	0.96	0.396	0.0152	0.364 [0.091–0.636]
Max Blob Eccentricity	0.96	0.354	0.0315	0.443 [0.125–0.727]
Age (Years)	NA	0.045	0.778	0.450 [0.182–0.75]
PSA (nG/mL)	NA	0.153	0.339	0.418 [0.0–0.909]
Prostate Volume (mL)	NA	0.174	0.271	0.464 [0.167–0.767]

**Abbreviations**: Max Blob eccentricity: Maximum Blob Eccentricity, Max Blob Vol: Maximum Blob Volume, Ave Blob Vol: Average Blob Volume, PSA: prostate serum antigen, R: Correlation Coefficient, AUC: Area Under the Curve, CI: 95% Confidence Interval, mL: milliliter, nG/mL: nanograms per milliliter.

**Table 3:** Summary of multivariate (logistic probability) fits of tumor eccentricity and tumor volume to ISUP grade (CsPCa).

ACE Threshold	Independent Variable 1	R1 (*p* Value)	Independent Variable 2	R2 (*p* Value)	R12 (F-Statistic Probability)	AUC [95% CI]
0.90	Max Blob Eccentricity	−0.145 (0.303)	Average Blob Volume	0.333 (0.029)	0.367 (0.0593)	0.542 [0.250–0.833]
0.90	Max Blob Eccentricity	−0.145 (0.551)	Max Blob Volume	0.333 (0.044)	0.345 (0.0847)	0.869 [0.636–1.0]
0.92	Max Blob Eccentricity	−0.101 (0.646)	Average Blob Volume	0.393 (0.0102)	0.399 (0.034)	0.640 [0.182–1.0]
0.92	Max Blob Eccentricity	−0.101 (0.780)	Max Blob Volume	0.348 (0.0310)	0.351 (0.0772)	0.959 [0.80–1.0]
0.94	Max Blob Eccentricity	−0.402 (0.028)	Average Blob Volume	0.419 (0.0190)	0.528 (0.0024)	0.896 [0.625–1.0]
0.94	Max Blob Eccentricity	−0.402 (0.037)	Max Blob Volume	0.371 (0.068)	0.484 (0.00716)	0.949 [0.778–1.0]
0.96	Max Blob Eccentricity	−0.354 (0.019)	Average Blob Volume	0.390 (0.011)	0.530 (0.00372)	0.084 [0–0.3]
0.96	Max Blob Eccentricity	−0.354 (0.018)	Max Blob Volume	0.396 (0.009)	0.536 (0.0032)	0.448 [0.182–0.727]

**Abbreviations**: Max Blob eccentricity: Maximum Blob Eccentricity, Max Blob Vol: Maximum Blob Volume, Ave Blob Vol: Average Blob Volume, PSA: prostate serum antigen, R1: Correlation Coefficient for Independent variable 1, R12: Correlation Coefficient for multivariate fit, AUC: Area Under the Curve, CI: 95% Confidence Interval, mL: milliliter, nG/mL: nanograms per milliliter.

## Data Availability

https://pi-cai.grand-challenge.org/ (accessed on 3 March 2023).

## Appendix A

### Appendix A.1. Adaptive Cosine Estimator (ACE)

The multispectral Supervised Target Detection (STDA) methods [10,11,12,24], specifically the Adaptive Cosine Estimator (ACE) algorithm (Equation (A1)), was applied to spatially registered BP-MRI. The ACE algorithm employs in-scene multispectral tumor signatures. *S*, the target (tumor) signature, is a 3-component vector (T2, ADC, High-B Value, see References [10,11,12,24]). *S*, the in-scene tumor signature, is selected from green voxels in a three-color display of the spatially-registered BP-MRI (red is ADC, green is High-B Value, blue is T2) [10,11,12,16,24]. The *S* vector, with the component *S_q_*, is the mean of the *T* target vector-voxels *x_p.q_* summed over *p* target voxels (identified as green in the composite color image) (Equation (A1)). The signature *S* is substituted into the ACE algorithm (Equation (A1)). The *m* vector is the background (normal prostate) 3-component vector. *CM* is the background covariance (3 × 3) matrix. Digital outlining of the normal prostate for all relevant slices on the spatially-registered BP-MRI [10,11,12,16,24] delineates a restrictive mask identifying the background voxels needed for m and *CM* computations. The *ACE* (*x_i_*) value at each voxel *x_i_* is given by Equation (A1).

(A1) $$S_{q}=\frac{1}{T}\sum_{p=1}^{T}x_{p.q},\;ACE(x_{i})=\frac{{\left(S-m\right)}^{T}CM^{-1}(x_{i}-m)}{\sqrt{\left[{\left(S-m\right)}^{T}CM^{-1}(S-m)][{\left(x_{i}-m\right)}^{T}CM^{-1}(x_{i}-m)\right]}}$$

*ACE* (*x_i_*) is the “whitened” cosine between the “whitened” test vector *x_i_* for voxel *i* and the “whitened” target signature *S* (Equation (A1)) [10,24]. The *ACE* (*x_i_*) value identifies whether *x_i_* is tumor or normal prostate tissue. The ACE algorithm employs the conical hyperspace decision surface to assess whether a voxel *x_i_* is a normal prostate or background (large angle, small cosine) or tumor or target (small angle or large cosine). The ACE scores range from −1.0 to 1.0. The identification of the voxel is set by the detection threshold given by the user based on previously examined data, such as optimal correlation with a grade.

### Appendix A.2. Eccentricity Calculation

Custom software (written in Python 3) computed the eccentricity and volume [12] for every labeled blob. Eigenvalues were computed from solving the eigenvalue equation for the two-dimensional moment of inertia *I* (Equation (A2)) and the standard defined matrix components *I_xx_*, *I_yy_*, *I_xy_*, and *I_yx_*. [32]

(A2) $$I=\left(\begin{array}{cc} I_{xx} & I_{xy}\\ I_{yx} & I_{yy} \end{array}\right)$$

Eccentricity uses the largest axis *L_k_* and transverse moments *S_k_* are eigenvalues from the moment of inertia matrix *I* for the kth blob. The eccentricity *E_k_* for the kth blob with a major axis *L_k_* and minor axis *S_k_* is given by (Equation (A3)).

(A3) $$E_{k}=\frac{L_{k}-S_{k}}{L_{k}+S_{k}}$$

Eccentricity values *E_k_* range from 0 < *E_k_* < 1. A spherical shape has an eccentricity *E_k_* of 0; a line has an eccentricity *E_k_* of 1.

### Appendix A.3. Tumor Volume Measurements, Supervised Target Detection

Previous work [11] described the procedure for estimating tumor volume using the supervised target detection algorithm or ACE algorithm. A threshold is applied to the ACE map (see Appendix A.1.). Voxels that exceed a threshold for the ACE scores are identified as a tumor, and normal tissues are identified by ACE scores falling below the threshold. The current study employed higher thresholds (>0.85) relative to an earlier study [11] that used thresholds ranging from 0.40 to 0.85 in 0.05 increments and found that 0.65 was optimal. The number voxels *N* within the *k*th blob exceeding the threshold were counted and converted to tumor volume *V_k_* (Equation (A4)) based on the MRI spatial resolution (~1.5 mm × ~1.5 mm) and slice separation (3 mm), resulting in a voxel volume (*r*~0.01 mL).

(A4) $$V_{k}=rN=r\sum_{i=1}^{N}\frac{x_{i}}{abs(x_{i})}.$$

### Appendix A.4. Univariate and Multivariate Fitting

The *ISUP* grade was determined through a pathological assessment of histology slides from a biopsy. The *ISUP* grades were fitted individually through a linear regression analysis with eccentricity (*Ecc*) or volume (*Vol*). The dependent variable, the *ISUP* grade, is univariately [14,15,26] linearly related to the independent measurements *Ecc* and *Vol*, i.e.:

(A5) $$ISUP=b_{const}+b_{Ecc}Ecc+\varepsilon$$

(A6) $$ISUP=b_{const}+b_{Vol}Vol+\varepsilon \;.$$

Optimal coefficients *b_const_*, *b_Ecc_*, and *b_Vol_* or constants, eccentricity coefficients, and volume coefficients, respectively, were chosen by minimizing the error e through the least squares formulation. In this study, Ecc includes eccentricity from the largest blob. The analysis filters out contributions from blobs smaller than 5 voxels (~10^−2^ mL) due to spatial registration errors inducing spurious detections. Vol or volume in this study includes the total tumor blob volume, average blob volume, and maximum blob volume. The fitness and probability that the fit departs from the null fit were assessed by computing the Pearson correlation coefficient R and *p*-value, respectively.

Specifically, the data tables only cite the Pearson correlation coefficient R, *p*-value, F-value and affiliated *p*-value.

In addition to univariate fits, a multivariate fit was applied to the *ISUP* grade, eccentricity, and volume measurements, i.e.:

(A7) $$ISUP=b_{const}+b_{Ecc}Ecc+b_{Vol}Vol+\varepsilon$$

### Appendix A.5. Receiver Operator Characteristic

The receiver operator characteristic curve characterizes [16,23] the binary classifier performance. The ROC curve plots the probability of target detection (or sensitivity) against the false alarm probability (or 1-specificity) for all threshold settings. In addition, a classifier’s accuracy can be evaluated by comparing the multivariable logistic regression fits with the pathologist’s CsPCA/CiPCa findings for each patient.

The vertical axis of the ROC curve (sensitivity) assesses the accuracy of identifying patients with clinically significant prostate cancer (CsPCa) by the logistic regression for a given threshold. The horizontal axis of the ROC curve (false alarm probability, 1-specificity) shows the relative accuracy of correctly identifying the patient’s status of clinically insignificant prostate cancer (CiPCa) for a given probability threshold. The ROC curve monotonically increases. Hypothetically, the best ROC curve value would be 100% target detection and 0% false alarm probability residing in the upper left corner for the ROC curve. The area under the curve (AUC) assesses the classifier where 0 < AUC < 1 and AUC = 0 (poor performance) and AUC = 1 (optimal performance).
